# Dietary glycemic index and load during pregnancy and offspring behavioral outcomes: exploring sex differences

**DOI:** 10.1007/s00431-025-06005-y

**Published:** 2025-02-06

**Authors:** Esther Cendra-Duarte, Josefa Canals, Nerea Becerra-Tomás, Javier Mateu-Fabregat, Mònica Bulló, Victoria Arija

**Affiliations:** 1 Universitat Rovira i Virgili, Unitat de Salut Pública i Epidemiologia Nutricional, Nutrition and Mental Health (NUTRISAM) Research Group, Reus, Spain; 2 Collaborative Group On Lifestyles, Nutrition, and Tobacco (CENIT), Institut d’Investigació en Atenció Primària IDIAP Jordi Gol, Institut Català de La Salut (ICS), Reus, Spain; 3 Institut d’Investigació Sanitària Pere Virgili (IISPV), Tarragona, Spain; 4 Universitat Rovira i Virgili, Centre de Recerca en Avaluació i Mesura de La Conducta (CRAMC), Department of Psychology, Tarragona, Spain; 5 Universitat Rovira i Virgili, Nutrition and Metabolic Health Research Group, Department of Biochemistry and Biotechnology, Reus, Spain; 6 Center of Environmental, Food and Toxicological Technology (TecnATox), Rovira i Virgili University, Reus, Spain; 7 CIBER Physiology of Obesity and Nutrition (CIBEROBN), Carlos III Health Institute, Madrid, Spain

**Keywords:** Child, Child development, Behavioral problems, Pregnancy, Pregnancy nutrition

## Abstract

Given the importance of carbohydrates during pregnancy and the limited evidence on the impact of its excessive intake on offspring neurodevelopment, this study aimed to assess the associations between maternal glycemic index (GI) and glycemic load (GL) during early and late pregnancy and behavior problems in 4-year-old children, considering potential sex-related differences in susceptibility to maternal diet. This observational study included 188 mother–child pairs from the ECLIPSES study. GI and GL were estimated from a validated food frequency questionnaire. Offspring behavior was assessed using the Child Behavior Checklist 1.5–5. Multivariable linear and logistic regression analyses were employed to assess the association between GI, GL, and child behavior. Children of mothers in the highest tertile of GL during the first trimester of pregnancy showed elevated scores of both internalizing (*β* = 5.77; 95% CI, 2.28–9.26) and externalizing (*β* = 3.95; 95% CI, 0.70–7.19) problems, including anxiety and depression problems, withdrawn, attention problems, aggressive behavior, and attention-deficit/hyperactivity problems, as well as total (*β* = 5.24; 95% CI, 1.71–8.77) and autism spectrum problems (*β* = 3.30; 95% CI, 1.11–5.50). Similarly, higher odd ratios were observed for internalizing (OR = 2.37; 95% CI, 1.09–5.18), externalizing (OR = 3.46; 95% CI, 1.49–8.00), and total problems (OR = 3.83; 95% CI, 1.68–8.71). These associations were more pronounced in girls. No associations were observed during the third trimester. Regarding GI, no associations were found for the evaluated outcomes in any of the trimesters.

*Conclusion*: These findings indicated that elevated maternal GL during the early pregnancy, but not later stages, was associated with adverse behavioral outcomes in offspring.

*Trial registration*: EUCTR-2012–005480-28, NCT03196882.

**Table:** 

**What is Known: ** • *Carbohydrate intake is important during pregnancy as glucose is the main energy source for an optimal fetal brain development.* • *Elevated prenatal glycemic index and glycemic load have been associated with adverse offspring outcomes but their impact on behavioral development remains insufficiently explored.*
**What is New:** • *A high maternal glycemic load during pregnancy may increase the risk of behavioral impairments in preschool-aged offspring.* • *Female offspring may be more vulnerable to behavioral disturbances to elevated maternal glycemic load during gestation.*

**What is Known: **

• *Carbohydrate intake is important during pregnancy as glucose is the main energy source for an optimal fetal brain development.*

• *Elevated prenatal glycemic index and glycemic load have been associated with adverse offspring outcomes but their impact on behavioral development remains insufficiently explored.*

**What is New:**

• *A high maternal glycemic load during pregnancy may increase the risk of behavioral impairments in preschool-aged offspring.*

• *Female offspring may be more vulnerable to behavioral disturbances to elevated maternal glycemic load during gestation.*

## Introduction

Maternal nutrition during pregnancy is crucial for fetal brain development [1]. Inadequate prenatal nutrient intake may increase the risk of neurodevelopmental defects and neuropsychiatric disorders in later life [2], such as autism spectrum disorder (ASD), attention-deficit/hyperactivity disorder (ADHD), anxiety, and depression [3, 4].

Pregnant women have greater nutritional requirements, particularly for carbohydrates, the main energy source for both mother and fetus [5]. Carbohydrates are essential for fetal tissue growth and cell development [6], and glucose, which passes through the placenta, is crucial for fetal brain development [7, 8]. However, the quantity and quality of carbohydrates are important, as they have an impact on maternal and child metabolism by affecting blood glucose and insulin levels [5, 9]. Glycemic index (GI) and glycemic load (GL) are commonly used dietary measures to quantify this. GI measures the post-prandial blood glucose response to carbohydrate-containing food [10]. GL represents both the glycemic index and the total carbohydrate intake, providing a more accurate indicator of the impact on postprandial glucose or blood glucose and insulin levels [10].

The relationship between maternal dietary GI and GL and offspring outcomes has been studied [11–14], with most research focusing on anthropometric outcomes [15–19], showing that higher maternal GI, and particularly GL, are linked to an increased risk of delivering large-for-gestational-age infants, [15–17] higher fetal and birth weights [16, 18], and higher adiposity in early childhood [17, 19]. However, to our knowledge, only one study has assessed the relationship between maternal GL and child behavior development [20], finding that children of mothers with higher prenatal GL were more likely to exhibit anxiety, impulsivity, and maladaptive and inhibition-related behaviors at age 2. Furthermore, in line with these results, studies investigating the impact of maternal elevated glucose levels on offspring development have reported an increased risk of externalizing problems and neurodevelopmental delays [21, 22].

Given the importance of carbohydrates on fetal neurodevelopment and the adverse effects of elevated maternal GI and GL on other offspring outcomes, further research is necessary to elucidate their impact on child’s behavior, as only one study in the USA has been conducted to date [20]. Therefore, this study aimed to assess the association between maternal GI and GL at the first and third trimesters of pregnancy and behavior problems in offspring at 4 years old. Additionally, potential sex-related differences in the susceptibility to maternal diet abnormalities were considered [23].

## Materials and methods

### Study population

The participants in this study were drawn from the ECLIPSES study [24], a community-based randomized controlled trial conducted on pregnant women in the province of Tarragona (Catalonia, Spain). Further details of the study can be found elsewhere [24]. The ECLIPSES study is registered at www.clinicaltrialsregister.eu (number EUCTR-2012–005480-28) and at www.clinicaltrials.gov (number NCT03196882).

**Fig. 1:**
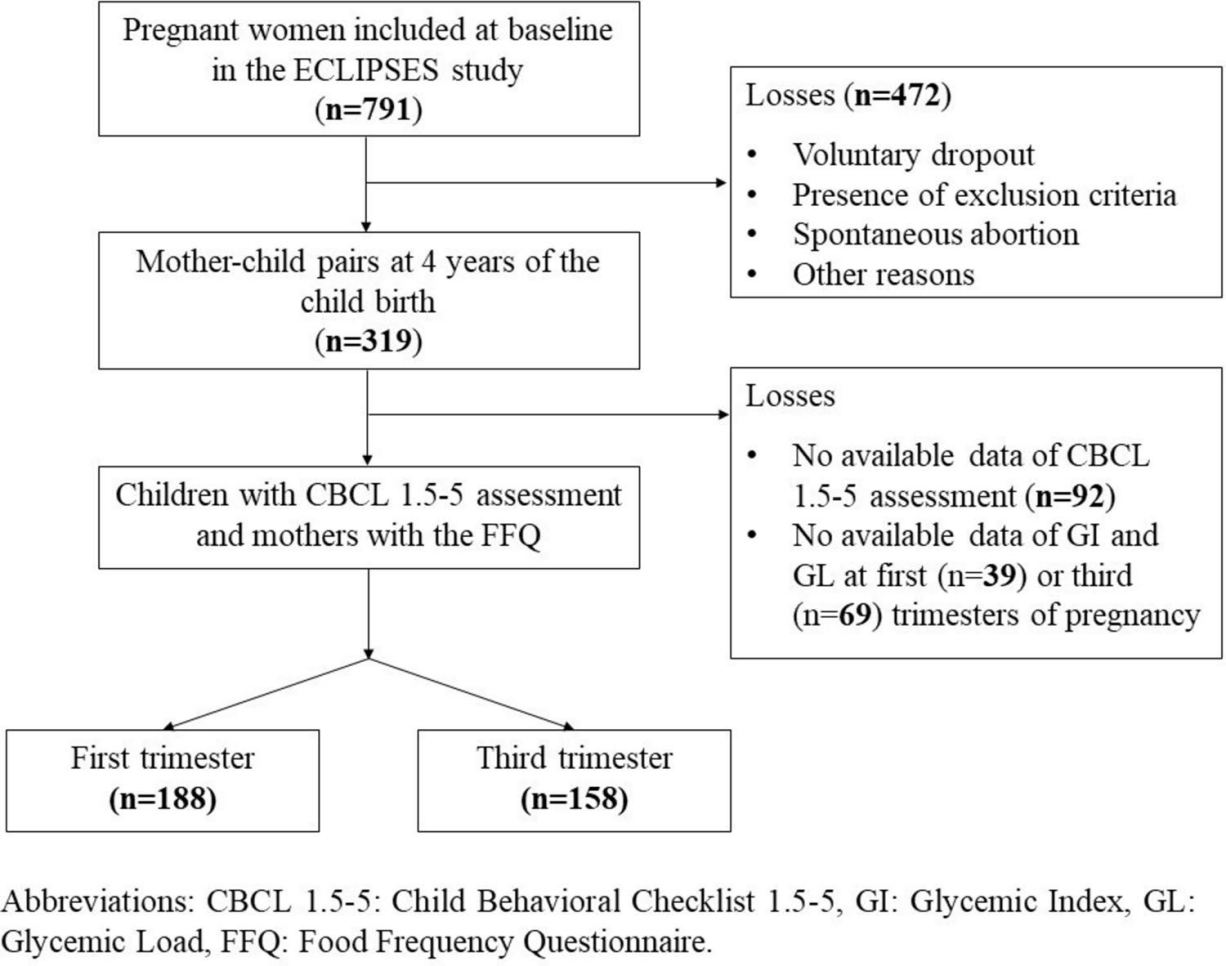
Flowchart of the study population

Eligible participants were healthy women over the age of 18 years with less than 12 weeks of gestation, with no anemia, and who were able to understand Catalan and Spanish languages and the characteristics of the study. None of the pregnant women included in the study had experienced gestational diabetes or other pathologies during pregnancy [24]. Of the 319 pairs of mothers and their children with information on neurodevelopment at 4 years of age, 188 mother–child pairs were included in our analyses, from whom we had data on the mother’s GI and GL during the first or third trimester of pregnancy and the child’s behavioral assessment at four years of age (Fig. 1).

### Maternal dietary glycemic index and glycemic load

Maternal GI and GL in the first and third trimester of gestation were calculated from a self-administrated validated food frequency questionnaire (FFQ) [25]. Pregnant women reported their weekly or monthly intake of 45 food items at weeks 12 and 36 of pregnancy. The daily consumption in grams/day of each item was calculated by multiplying the reported frequency by the average daily consumption observed in the population [26]. The energy intake and macronutrients were estimated with the REGAL (Répertoire Général des Aliments) food composition table [27], complemented by the Mataix Verdú Spanish food composition table [28]. The GI values were extracted from the international tables of GI and GL values [29], with glucose as the reference. In the absence of data for specific food item, a mean was calculated for similar food items included in the FFQ. The dietary GL was estimated by multiplying the glycemic index of each food item by the available carbohydrates (in grams) per serving and the amount consumed, summing these values for all food items, and then dividing the total by 100. The dietary GI was calculated by multiplying the GI of each food item by the available carbohydrates (in grams) per serving, summing these values, and then dividing the result by the total carbohydrate consumption [20, 30, 31].

### Child behavioral assessment

Children’s behavioral problems were evaluated by means of the Child Behavior Checklist for ages 1.5 to 5 years (CBCL 1.5–5) [32]. The CBCL 1.5–5 is a tool completed by the parents that includes 99 items rated on a three-point scale (not true, sometimes true, very true), reflecting the child’s emotional, behavioral, and social problems as perceived by parents. The assessments produce 6 syndrome scales and 5 DSM (Diagnostic and Statistical Manual of Mental Disorders)-oriented scales, in addition to 3 broad-band scales (externalizing, internalizing, and total problems). Internalizing scale comprises the syndromic subscales of emotional reactivity, anxious/depressed, somatic complaints, and withdrawn. Externalizing scales comprise the subscales of aggressive behavior and attentional problems. Total problems scale comprises all syndromic subscales and provides a comprehensive measure of the emotional-behavioral well-being or difficulties of the children. *T*-scores were employed, with scores between 65 and 69 classified as borderline range and scores equal to or above 70 as clinical range for both syndrome and DSM-oriented scales. For broad-band scales, the borderline range was considered between 60 and 64 points, while a score equal to or greater than 65 indicated a clinical range. For the categorical analyses, the borderline and clinical ranges were considered together as a single category for scoring as a clinical score. The Spanish version of the CBCL1.5–5 has demonstrated satisfactory moderate to good internal consistency and a high capacity to differentiate between disruptive and internalizing disorders in preschool children [33].

### Other variables

Baseline information was collected at the first trimester visit, including maternal age and educational level (primary, secondary, or university studies). At each trimester visit, data were also collected on maternal height and weight to calculate their body mass index (BMI) and the gestational weight gain (GWG) (difference between the third and first trimester weights). Additionally, we assessed if participants met the Institute of Medicine (IOM) recommendations for pregnancy weight gain [34]. Lifestyle habits were evaluated using the Fagerström test to assess smoking [35], the FFQ for alcohol consumption [25], and the short version of the International Physical Activity Questionnaire (IPAQ-S) for physical activity [36]. Anxiety symptoms were evaluated by the State-Trait Anxiety Inventory (STAI) [37], which assesses two different anxiety concepts, “state” and “trait”, and we focused our analyses on state anxiety scores. At the 4-year follow-up visit, mothers completed the validated Spanish version [38] of the Goldberg Anxiety and Depression Scale [39].

Child data included sex, birth weight, gestational age, and maternal-child attachment, assessed with the Parenting Stress Index (PSI) test [40, 41]. At 4 years of age, either exclusive or mixed breastfeeding duration, age and dietary assessment were recorded. Dietary assessment was recorded using a validated short FFQ for children [42]. A diet quality index was then calculated based on the Spanish Diet Quality Index (SDQI) [43], which categorizes ten food groups according to their nutritional quality and compares real consumption with recommended servings [44]. A score is assigned to each group of food, ranging from 0 (does not meet the recommendations) to 10 (meets the recommendations), and the sum of these scores allows for a final score to be calculated, ranging from 0 to 100 points.

### Statistical analyses

The first and third trimester GI and GL were energy-adjusted using the residual method [45]. First trimester energy-adjusted GI and GL variables were divided into tertiles. The cut-off points of the tertiles established at the first trimester were also used to create the three-categorical variables for energy-adjusted GI and GL in the third trimester. Subsequently, dichotomous variables were created to assess the differences between high (Tertile 3) and low-middle (Tertile 1 and Tertile 2) prenatal GI and GL and child behavior problems. Baseline characteristics of the study participants were expressed as means (± standard deviations) for quantitative variables and frequency (percentages) for categorical variables. The Shapiro–Wilk test was employed to assess the normality of continuous data. Multivariable linear and logistic regression models were performed to assess the relationship between maternal GI and GL (high vs low-middle) in the first and third trimester and children’s behavioral problems. All models were adjusted for the following potential maternal and child confounding factors: mother’s age (< 35 years or ≥ 35 years), educational level (primary/secondary studies or university studies), first trimester BMI (kg/m^2^), GWG (met or no the recommendations), intervention group (40 mg vs 80 mg of iron or 40 mg vs 20 mg of iron), smoking (non-smoker/ex-smoker or smoker), anxiety at first or third trimester (score), energy intake at first or third trimester (kilocalories/day), physical activity at first or third trimester (METs/min/week), gestational age (weeks), breastfeeding duration (< 6 months or ≥ 6 months), maternal and child attachment (score), maternal anxiety or depression at 4 years visit (no or yes), child’s sex (boy or girl), child’s birth weight (low birth weight or normal birth weight), and child’s diet quality (score). The following potential confounders had missing values: anxiety at the first trimester (5.85%), anxiety at the third trimester (10.12%), physical activity at the third trimester (8.22%), gestational weight gain (24.46%), breastfeeding duration (3.19%), maternal and child attachment (1.59%), child’s diet quality (2.17%), maternal anxiety or depression at 4 years visit (1.06%), and child’s birth weight (3.72%). Missing data were imputed to the median value for quantitative variables and to the highest frequency category for qualitative variables [46].

A multivariable linear regression analyses for GL were also conducted stratifying by child’s sex. GL was selected over GI as it is considered a more accurate and informative measure of the impact of carbohydrate quality and quantity on maternal glucose levels [47], and fetal sensitivity to changes in those levels differs depending on the sex of the child [48].

The analyses were conducted using IBM SPSS Statistics for Windows, version 29.0 (Armonk, NY: IBM Corp) and R statistical package version 4.3.3 (R foundation for statistical computing, Vienna, Austria; version 4.2.2). Statistical significance was set at a *p*-value < 0.05.

## Results

Table 1 presents the study population characteristics. The mean age of the mothers was 31.8 ± 4.4 years, with 46.8% having university studies. Their mean first trimester BMI was 24.9 ± 4.6 kg/m^2^, being 13.3% in the obesity range, while the mean GWG was 10.1 ± 3.5 kg, with over 60% not meeting the IOM recommendations [34]. During pregnancy, 16.0% reported smoking and 1.6% alcohol intake. Participants were moderately active, with energy intakes of 2005.5 ± 500.8 and 2108.6 ± 472.2 kcal/day at the first and third trimesters, respectively. At first trimester, pregnant women had a mean GI of 61.9 ± 9.8, a mean GL of 104.22 ± 38.59, and a mean carbohydrate intake of 169.84 ± 59.89 g/day, which are close to the recommended intake values (175 g/day) [49] and very similar to the values of the third trimester: mean GI of 61.8 ± 9.1, a mean GL of 98.0 ± 29.8, and a mean carbohydrate intake of 159.9 ± 48.5. Mothers exhibited below-average anxiety scores, but at the 4-year follow-up visit, almost 63% had anxiety or depression. Regarding the children (51.1% girls), the mean age was 4.3 ± 0.3 years, with a mean birth weight of 3297.8 ± 436.2 g and a mean gestational age of 39.5 ± 1.5 weeks. Children had a mean breastfeeding duration of 10.8 ± 12.3 months, with 59% having a duration of 6 months or more. Maternal and child attachment scores were within the average range and child diet quality needed improvement. The mean CBCL 1.5–5 scale scores were within the normal range. However, 35.4% of girls and 41.3% of boys exhibited clinical scores (borderline and clinical ranges) for internalizing and externalizing problems, respectively. The characteristics of our participants (including age, BMI, educational level, and lifestyle habits) were not statistically significantly different from those of the non-included participants.

**Table 1:** Characteristics of the participants

Sample size, *n* = 188			
**Maternal characteristics**			
Age (years)	31.8 ± 4.4		
First trimester BMI (kg/m^2^)	24.9 ± 4.6		
Obesity	25 (13.3)		
Gestational weight gain (kg)	10.1 ± 3.5		
Meet the recommendations	72 (38.3)		
Do not meet the recommendations	116 (61.7)		
Educational level			
Primary or secondary studies	100 (53.2)		
University studies	88 (46.8)		
Smoke during pregnancy (yes)	30 (16.0)		
Alcohol during pregnancy (yes)	3 (1.6)		
Physical activity at the first trimester (METs/min/week)	1939.5 ± 2415.1		
Physical activity at the third trimester (METs/min/week)*	2005.8 ± 2231.6		
Energy intake at the first trimester (kcal/day)	2005.5 ± 500.8		
Energy intake at the third trimester (kcal/day)*	2108.6 ± 472.2		
Carbohydrates at the first trimester (g/day)	169.8 ± 59.9		
Carbohydrates at the third trimester (g/day)*	159.9 ± 48.5		
Glycemic index at the first trimester (score)	61.9 ± 9.8		
Glycemic index at the third trimester (score)*	61.8 ± 9.1		
Glycemic load at the first trimester (score)	104.2 ± 38.6		
Glycemic load at the third trimester (score)*	98.0 ± 29.8		
Anxiety at the first trimester (score)	17.0 ± 7.8		
Anxiety at the third trimester (score)*	18.8 ± 7.7		
Anxiety and depression at 4-year visit (yes)	118 (62.8)		
**Children characteristics**			
Age (years)	4.3 ± 0.3		
Sex (girl)	96 (51.1)		
Birth weight (grams)	3297.8 ± 436.2		
Apgar (score)	9.59 (0.39)		
Gestational age (weeks)	39.5 ± 1.5		
Type of delivery			
Eutocic	128 (68.1)		
Dystocic	60 (31.9)		
Breastfeeding duration (months)	10.8 ± 12.3		
< 6 months	77 (41.0)		
≥ 6 months	111 (59.0)		
Maternal and child attachment (score)	51.8 ± 5.4		
Diet quality (score)	61.5 ± 10.7		
Behavioral assessment (CBCL 1.5–5) (score)	Mean ± SD	*n* (%) of girls^a^	*n* (%) of boys^b^
Syndrome scales			
Emotionally reactive	57.2 ± 8.9	14 (14.6)	20 (21.7)
Anxious/depressed	56.1 ± 7.4	14 (14.6)	13 (14.1)
Somatic complaints	55.5 ± 6.9	15 (15.6)	13 (14.1)
Withdrawn	58.1 ± 7.7	8 (8.3)	25 (27.2)
Attention problems	57.7 ± 7.1	13 (13.5)	34 (37.0)
Aggressive behavior	55.5 ± 7.4	5 (5.2)	15 (16.3)
Broad-band scales			
Internalizing problems	54.9 ± 12.0	34 (35.4)	36 (39.1)
Externalizing problems	53.5 ± 10.9	19 (19.8)	38 (41.3)
Total problems	54.6 ± 12.2	25 (26.0)	37 (40.2)
DSM-oriented scales			
Depressive problems	56.6 ± 7.3	9 (9.4)	22 (23.9)
Anxiety problems	57.5 ± 8.3	18 (18.8)	18 (19.6)
Autism spectrum problems	57.6 ± 7.6	15 (15.6)	27 (29.3)
Attention deficit/hyperactivity problems	57.3 ± 7.7	12 (12.5)	26 (28.3)
Oppositional defiant problems	54.8 ± 6.8	7 (7.3)	16 (17.4)

Values are expressed in terms of mean ± standard deviation (SD) or frequency (*n*) and percentages (%) as appropriate. *BMI* body mass index, *CBCL 1.5–5* Child Behavior Checklist 1.5–5, *DSM* Diagnostic and Statistical Manual of Mental Disorders

^*^Sample size, *n* = 158

^a^Girls (*n* = 96) who had clinical scores (≥ 65 for syndrome and DSM-oriented scales, and scores ≥ 60 for broad-band scales)

^b^Boys (*n* = 92) who had clinical scores (≥ 65 for syndrome and DSM-oriented scales, and scores ≥ 60 for broad-band scales)

**Fig. 2:**
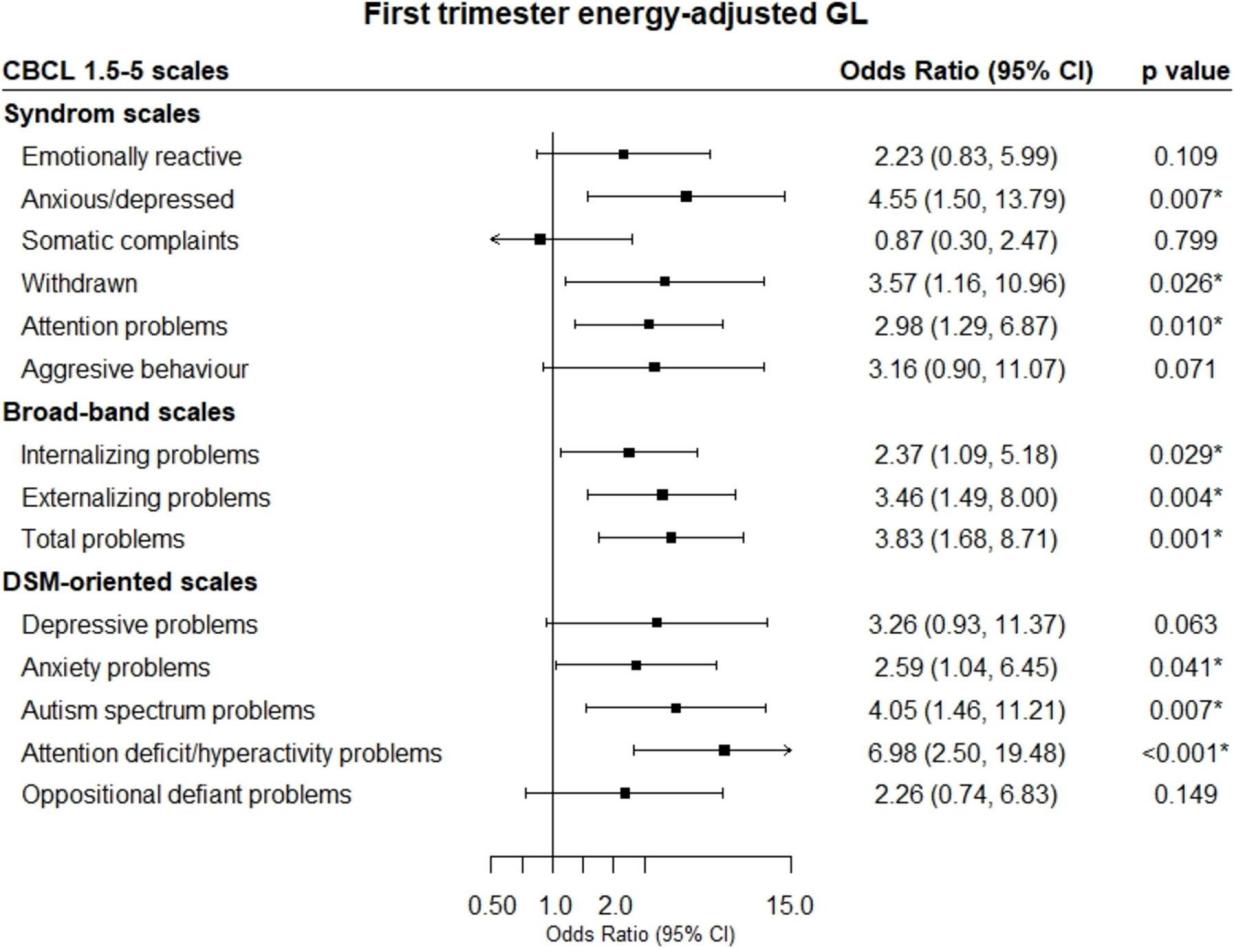
Multivariable-adjusted odds ratio of behavioral problems of children assessed with CBCL 1.5–5 and maternal first trimester energy-adjusted GL. CBCL 1.5–5, Child Behavior Checklist 1.5–5; CI, confidence interval; GL, glycemic load. Models were adjusted for: maternal age, educational level, first trimester BMI, intervention group, smoking, anxiety state at the first trimester, energy intake at the first trimester, physical activity at the first trimester, gestational weight gain, gestational age, breastfeeding duration, maternal and child attachment, maternal anxiety at 4 years, child’s sex, child’s birth weight, and child’s diet quality. The diamonds represent odds ratios and the whisker plots represent 95% CIs. *p-values are statistically significant

The associations between maternal energy-adjusted GI and GL and children’s behavioral problems are presented in Table 2. Compared to children of mothers with low-middle energy-adjusted GL, children of mothers with high GL in the first trimester of pregnancy had higher scores for anxious/depressed, withdrawn, attention problems, aggressive behavior, internalizing problems, externalizing problems, total problems, DSM-depressive problems, DSM-anxiety problems, DSM-autism spectrum (DSM-ASD) problems and DSM-attention deficit/hyperactivity (DSM-ADHD) problems on the CBCL 1.5–5 scales (*β* coefficients ranging from 2.29 to 5.77). Furthermore, in multivariable logistic regression models, high maternal GL was associated with an increased probability of having clinical scores for anxious/depressed, withdrawn, attention problems, internalizing problems, externalizing problems, total problems, DSM-anxiety problems, DSM-ASD problems, and DSM-ADHD problems, with odds ratios ranging from 2.37 to 6.98 (Fig. 2). In contrast, no statistically significant associations were observed between maternal GL in the third trimester, nor for GI in both trimesters, and child behavior problems.

**Table 2:** Multivariable linear regression between the third tertile of maternal energy-adjusted glycemic index and glycemic load at first and third trimesters of pregnancy and behavioral problems of the child at 4 years of age

CBCL 1.5–5 scales	Energy-adjusted high glycemic index				Energy-adjusted high glycemic load			
	First trimester (*n* = 188)		Third trimester (*n* = 158)		First trimester (*n* = 188)		Third trimester (*n* = 158)	
	*β* (95% CI)	*p*-value	*β* (95% CI)	*p*-value	*β* (95% CI)	*p*-value	*β* (95%CI)	*p*-value
**Syndrome scales**								
Emotionally reactive	− 1.13 (− 3.69, 1.42)	0.383	− 0.46 (− 3.41, 2.47)	0.753	2.03 (− 0.70, 4.77)	0.145	− 0.29 (− 2.97, 3.56)	0.859
Anxious/depressed	− 0.19 (− 2.41, 2.02)	0.863	0.70 (− 1.96, 3.36)	0.603	3.76 (1.45, 6.08)	0.002*	1.31 (− 1.63, 4.26)	0.380
Somatic complaints	− 0.07 (− 2.02, 1.88)	0.942	− 1.78 (− 4.00, 0.43)	0.113	1.19 (− 0.89, 3.28)	0.361	− 0.36 (− 2.84, 2.11)	0.772
Withdrawn	− 1.59 (− 3.79, 0.60)	0.155	0.80 (− 1.89, 3.50)	0.558	2.77 (0.43, 5.11)	0.020*	0.75 (− 2.24, 3.75)	0.621
Attention problems	− 0.70 (− 2.77, 1.36)	0.501	1.42 (− 0.88, 3.73)	0.224	3.18 (1.01, 5.35)	0.004*	0.48 (− 2.09, 3.06)	0.712
Aggressive behavior	− 1.54 (− 3.65, 0.56)	0.149	0.72 (− 1.80, 3.25)	0.573	2.29 (0.04, 4.54)	0.046*	− 0.75 (− 3.56, 2.05)	0.597
**Broad-band scales**								
Internalizing problems	− 1.63 (− 4.97, 1.69)	0.334	− 0.95 (− 4.84, 2.94)	0.630	5.77 (2.28, 9.26)	0.001*	1.39 (− 2.93, 5.71)	0.525
Externalizing problems	− 1.91 (− 4.97, 1.14)	0.218	0.87 (− 2.78, 4.54)	0.636	3.95 (0.70, 7.19)	0.017*	− 1.16 (− 5.22, 2.90)	0.573
Total problems	− 1.34 (− 4.70, 2.01)	0.431	− 0.64 (− 4.63, 3.34)	0.749	5.24 (1.71, 8.77)	0.004*	0.73 (− 3.70, 5.16)	0.745
**DSM-oriented scales**								
Depressive problems	− 1.20 (− 3.24, 0.83)	0.245	− 0.86 (− 3.26, 1.54)	0.480	2.33 (0.16, 4.50)	0.035*	1.85 (− 0.80, 4.51)	0.170
Anxiety problems	− 0.19 (− 2.26, 2.65)	0.876	− 1.12 (− 3.95, 1.71)	0.435	3.43 (0.84, 6.02)	0.010*	0.58 (− 2.57, 3.74)	0.714
Autism spectrum problems	− 0.51 (− 2.60, 1.57)	0.626	− 0.08 (− 2.34, 2.52)	0.942	3.30 (1.11, 5.50)	0.003*	1.20 (− 1.48, 3.90)	0.378
Attention-deficit/hyperactivity problems	− 0.01 (− 2.22, 2.25)	0.990	1.63 (− 0.99, 4.25)	0.222	4.04 (1.72, 6.37)	< 0.001*	0.01 (− 2.92, 2.94)	0.996
Oppositional defiant problems	− 1.62 (− 3.63, 0.38)	0.112	1.38 (− 1.02, 3.78)	0.258	1.97 (− 0.18, 4.12)	0.073	− 0.83 (− 3.50, 1.84)	0.539

*CBCL 1.5–5* Child Behavior Checklist 1.5–5, *CI* confidence interval, *DSM* Diagnostic and Statistical Manual of Mental Disorders. Adjusted for: maternal age, educational level, first trimester BMI, intervention group, smoking, anxiety state at 1st or 3rd trimester, energy intake at 1st or 3rd trimester, physical activity at 1st or 3rd trimester, gestational weight gain, gestational age, breastfeeding duration, maternal and child attachment, maternal anxiety at 4 years, child’s sex, child’s birth weight, and child’s diet quality. *p-value < 0.05

*β* coefficients are for high (Tertile 3) vs low-middle (Tertile 1 and Tertile 2) energy-adjusted glycemic index and glycemic load

Table 3 shows the association between maternal GL and child behavior problems stratified according to the child’s sex. Regarding girls, it was observed that those whose mothers were in the third tertile of energy-adjusted GL during the first trimester of gestation exhibited higher scores for anxious/depressed, aggressive behavior, internalizing problems, externalizing problems, total problems, DSM-depressed problems, DSM-anxiety problems, and DSM-ADHD problems (*β* coefficients ranging from 2.93 to 7.05). In relation to boys, high first trimester maternal energy-adjusted GL was only related to increased DSM-ADHD problems (*β* = 3.88; 95% CI from 0.38 to 7.38). In contrast, in the third trimester, there was no association between energy-adjusted GL and CBCL 1.5–5 scales in either boys or girls. Although no significant interaction was observed between child sex and GL, sex-specific analyses were conducted due to biological plausibility.

**Table 3:** Multivariable linear regression between the third tertile of maternal energy-adjusted glycemic load at first and third trimester of pregnancy and behavioral problems in girls and boys at 4 years of age

CBCL 1.5–5 scales	Girls				Boys			
	First trimester (*n* = 96)		Third trimester (*n* = 76)		First trimester (*n* = 92)		Third trimester (*n* = 82)	
	*β* (95% CI)	*p*-value	*β* (95% CI)	*p*-value	*β* (95% CI)	*p*-value	*β* (95% CI)	*p*-value
**Syndrome scales**								
Emotionally reactive	3.34 (− 0.54, 7.23)	0.091	4.13 (− 0.91, 9.19)	0.107	− 0.38 (− 4.65, 3.88)	0.857	− 3.54 (− 8.89, 1.79)	0.189
Anxious/depressed	5.99 (2.68, 9.31)	< 0.001*	4.28 (− 0.33, 8.90)	0.068	0.07 (− 3.46, 3.60)	0.968	− 0.73 (− 5.42, 3.94)	0.753
Somatic complaints	0.32 (− 2.85, 3.50)	0.839	− 0.46 (− 4.42, 3.49)	0.815	1.52 (− 1.65, 4.71)	0.341	0.90 (− 3.00, 4.80)	0.647
Withdrawn	1.14 (− 2.05, 4.35)	0.477	2.84 (− 1.37, 7.05)	0.182	2.50 (− 1.28, 6.29)	0.191	− 2.55 (− 7.6, 2.55)	0.321
Attention problems	3.05 (− 0.14, 6.26)	0.061	3.28 (− 0.53, 7.10)	0.090	2.83 (− 0.24, 5.92)	0.071	− 2.96 (− 6.72, 0.79)	0.120
Aggressive behavior	2.93 (0.29, 5.58)	0.030*	3.02 (− 0.37, 6.43)	0.080	2.14 (− 1.87, 6.16)	0.291	− 4.91 (− 9.93, 0.10)	0.055
**Broad-band scales**								
Internalizing problems	5.93 (0.67, 11.19)	0.027*	4.93 (− 2.11, 11.99)	0.166	3.41 (− 1.79, 8.61)	0.195	− 0.78 (− 7.57, 6.01)	0.819
Externalizing problems	5.68 (1.10, 10.26)	0.016*	3.72 (− 2.32, 9.76)	0.223	2.63 (− 2.50, 7.78)	0.310	− 5.79 (− 12.22, 0.63)	0.077
Total problems	7.05 (2.01, 12.09)	0.007*	5.26 (− 1.49, 12.02)	0.125	2.28 (− 3.28, 7.85)	0.416	− 2.52 (− 9.62, 4.58)	0.481
**DSM-oriented scales**								
Depressive problems	3.42 (0.32, 6.52)	0.031*	3.49 (− 0.14, 7.14)	0.060	0.51 (− 3.00, 4.03)	0.772	0.75 (− 4.00, 5.51)	0.753
Anxiety problems	5.35 (1.59, 9.10)	0.006*	1.55 (− 3.20, 6.31)	0.515	0.47 (− 3.44, 4.40)	0.808	0.97 (− 4.12, 6.06)	0.705
Autism spectrum problems	2.91 (− 0.14, 5.97)	0.062	2.28 (− 1.82, 6.39)	0.271	2.15 (− 1.31, 5.62)	0.219	− 0.23 (− 4.03, 4.50)	0.912
Attention-deficit/hyperactivity problems	4.56 (1.31, 7.82)	0.007*	2.81 (− 1.54, 7.17)	0.201	3.88 (0.38, 7.38)	0.030*	− 3.94 (− 8.46, 0.58)	0.086
Oppositional defiant problems	2.44 (− 0.22, 5.11)	0.072	2.90 (− 0.44, 6.25)	0.087	2.20 (− 1.52, 5.93)	0.243	− 4.52 (− 9.30, 0.26)	0.063

*CBCL 1.5–5* Child Behavior Checklist 1.5–5, *CI* confidence interval, *DSM* Diagnostic and Statistical Manual of Mental Disorders. Adjusted for: maternal age, educational level, first trimester BMI, intervention group, smoking, anxiety state at 1st or 3rd trimester, energy intake at 1st or 3rd trimester, physical activity at 1st or 3rd trimester, gestational weight gain, gestational age, breastfeeding duration, maternal and child attachment, maternal anxiety at 4 years, child’s birth weight and child’s diet quality. *p-value < 0.05

*β* coefficients are for high (Tertile 3) vs low-middle (Tertile 1 and Tertile 2) energy-adjusted glycemic load

## Discussion

The principal findings of this study showed that high energy-adjusted GL during early pregnancy was associated with adverse behavioral outcomes of the offspring at 4 years of age, most notably in girls. In contrast, no associations were observed with respect to maternal energy-adjusted GI, as this metric only assesses the glycemic response independently of carbohydrate quantity. This distinction, which is captured by GL, may explain the observed effect.

Supporting our findings, one previous study [20] evaluated maternal GL at the periconceptional period, focusing on children up to 2 years of age. Their findings indicated that high maternal GL was negatively associated with child neurodevelopment and temperament, as offspring were more prone to have internalizing and externalizing problems, including anxiety, inhibition, and maladaptive behaviors [20]. In the context of this association, the influence of carbohydrates from the maternal diet, particularly sugars, on the offspring’s behavior has also been investigated. The results of studies conducted on mice [50, 51] and humans [52, 53] suggest that an elevated maternal intake of sugars (i.e., fructose, sucrose, and sugar-/artificially sweetened beverages) during pregnancy is associated with an elevated risk of anxiety [51] and hyperactive behavior [50, 52], as well as a delayed onset of social and emotional development in the offspring [53].

In addition to this, other research [21, 22, 54–60] has investigated the impact of altered glucose levels during pregnancy, including hyperglycemia and gestational diabetes mellitus (GDM), on infant behavioral development. The findings indicated a positive association between maternal glucose abnormalities and neurobehavioral delays [54, 55], particularly externalizing problems such as ADHD and ASD [21, 22, 56–58], in offspring up to 5 years of age. However, some studies reported no association between GDM and behavioral development in children in early childhood [59], as well as in mid-childhood [60], which may suggest that early effects may attenuate over time.

The results of our study can be explained by certain mechanisms. Carbohydrates play an important role in providing energy and controlling glycemia and insulin metabolism [61], with maternal glucose levels being largely influenced by carbohydrate consumption [18, 62]. As maternal glucose crosses the placental [63], fluctuating changes in glucose levels during pregnancy can lead to alterations in the placenta [64, 65] and the metabolism and development of the fetus [21, 54], particularly its brain [66]. The brain requires an adequate supply of glucose to perform important processes for physiological function such as the generation of ATP, neuronal cellular maintenance, and the generation of neurotransmitters [7]. Excess glucose or hyperglycemia can lead to the onset of processes such as oxidative stress, hypoxia, hyperinsulinemia, and decreased iron levels in the fetus [57, 67–69]. These processes can cause damage to the brain and cellular DNA, including the central nervous system [68], which can result in neurodevelopmental disturbances and psychiatric problems in the offspring later in life [69]. Moreover, hyperglycemia can result in systemic inflammation [70], with pro-inflammatory cytokines crossing the placenta [71], affecting development, functionality, and neurogenesis in the hippocampus [67, 68, 70], a brain area involved in emotional, behavioral, and memory processing [72] and particularly susceptible to alterations in glucose levels [68].

Our findings revealed that high energy-adjusted GL in the first trimester, but not in the third, was associated with behavioral problems in offspring. Another previous study found comparable findings regarding the differences in the results of the impact of GL between trimesters in relation to child adiposity, showing that high GL in the first, but not in the third trimester of pregnancy was associated with fat mass at 4 and 6 years of age [19]. Although some neurodevelopmental processes continue after birth [73], the early stages of pregnancy represent a period of vulnerability for embryonic brain development, with important events such as neural tube formation, neurulation, cell proliferation, and cell migration occurring during this time [74–76]. In fact, research has identified the first trimester of pregnancy as a period of risk for the onset of neurodevelopmental disorders such as ADHD or ASD, due to the brain’s higher sensitivity to environmental factors, especially nutritional imbalances, during this time [74, 75, 77].

The adverse effect of higher energy-adjusted GL during the first trimester of pregnancy on offspring behavior was more pronounced in girls. Alick et al. [20] also observed that the highest tertile of maternal GL was associated with increased externalizing problems in boys, while in girls, only internalizing problems showed a relationship. Previous research states that female fetuses might be more vulnerable to metabolic alterations and to an excess of maternal glucose and sugars [48, 78, 79]. It is postulated that the underlying mechanisms may involve differential growth factors or gene expressions in placentas according to sex [79, 80]. Further research is required on both sexes to gain a deeper understanding of how changes in maternal diet during pregnancy influence behavioral patterns in boys and girls separately.

The principal strengths of this study are as follows: To the best of our knowledge, this is the first study to examine both GI and GL in early and late pregnancy with behavioral outcomes in preschool-aged children; a comprehensive data set was gathered in both pregnancy and childhood, thereby enabling the control of a variety of potential confounding factors in the analyses. However, it is important to consider some limitations when interpreting these findings. Firstly, the behavioral assessment was based on parent-reported data rather than clinical observation and without consideration of data from other informants. Nevertheless, it was conducted using a validated and widely recognized test in this field, ensuring the reliability of the results. Secondly, the final sample size of mother–child pairs that completed the assessments was reduced, which may limit the generalizability and statistical power. Thirdly, there is a possibility of recall bias and measurement error in the dietary estimation derived from the self-administered FFQ, which is common to all studies that evaluate dietary intake through this method. However, the FFQ used in this study was validated in our population. Fourthly, as this is an observational study, causality cannot be established. Lastly, although we controlled for many potential confounders, due to the observational nature of the study, there remains the possibility of residual confounding (e.g. genetic predisposition to some neuropsychiatric disorders).

In conclusion, we observed that higher energy-adjusted GL during early pregnancy may be associated with an increased risk of externalizing and internalizing behavioral problems in children, particularly in girls, at the age of 4 years. No significant associations were found for the GI. Considering that this is the first study to report these findings, further research is required to confirm this association and the potential sex differences in the impact on neurodevelopment.

## Data Availability

No datasets were generated or analysed during the current study.

## Abbreviations

ADHD: Attention-deficit/hyperactivity disorder
ASD: Autism spectrum disorder
BMI: Body mass index
CBCL 1.5–5: Child Behavior Checklist 1.5–5
DSM: Diagnostic and Statistical Manual of Mental Disorders
DSM-ADHD: DSM-attention deficit/hyperactivity
DSM-ASD: DSM-autism spectrum
FFQ: Food frequency questionnaire
GDM: Gestational diabetes mellitus
GI: Glycemic index
GL: Glycemic load
GWG: Gestational weight gain
IOM: Institute of Medicine
IPAQ-S: International Physical Activity Questionnaire
PSI: Parenting Stress Index
SDQI: Spanish Diet Quality Index
STAI: State-Trait Anxiety Inventory
